# Depression and Anxiety among Patients with Epilepsy and Multiple Sclerosis: UAE Comparative Study

**DOI:** 10.1155/2015/196373

**Published:** 2015-10-21

**Authors:** Taoufik Alsaadi, Khadija El Hammasi, Tarek M. Shahrour, Mustafa Shakra, Lamya Turkawi, Wassim Nasreddine, Mufeed Raoof

**Affiliations:** ^1^Department of Neurology, Sheikh Khalifa Medical City (SKMC), Abu Dhabi 51900, UAE; ^2^Department of Psychiatry, Sheikh Khalifa Medical City (SKMC), Abu Dhabi 51900, UAE; ^3^Department of Neurology, American University of Beirut Medical Center, Beirut 1107, Lebanon

## Abstract

Depression and anxiety are highly prevalent in patients with epilepsy (PWE), with prevalence rates ranging from 20% to 55%. The cause of this increased rate is multifactorial. Depression and epilepsy are thought to share the same pathogenic mechanism. Anxiety, on the other hand, seems to have a prevalence rate among PWE comparable to, or even higher than, those reported for depression, and it is closely linked to epilepsy. To test this hypothesis, we screened for depression and anxiety 186 and 160 patients attending the epilepsy and MS clinics, respectively, using standardized screening tools to determine the rates of both depression and anxiety, comparing these rates to 186 age, sex matched controls. Among the three groups, only patients with epilepsy were at increased risk of having depression (OR = 1.9), whereas anxiety was not. This finding could point to the shared pathogenic mechanisms hypothesis between depression and epilepsy.

## 1. Introduction

Epilepsy has been associated with increased risk of various psychiatric disorders with prevalence rates ranging between 12% and 41%; this variation in rates is largely due to methodological differences among the studies [1–5]. Depression and anxiety are among the most frequent psychiatric comorbidities in patients with epilepsy (PWE), with a prevalence of depression estimated between 11% and 60% in patients with recurrent seizures [6, 7]. Indeed, a recently published meta-analysis of several well designed studies of depression in PWE concluded that the risk of depression increases several folds in PWE, when compared to the general population [8]. On the other hand, anxiety in PWE has not been well studied or given the attention it deserves [9]. Surprisingly though, anxiety seems to have prevalence rates among PWE comparable to, or even higher than, those reported for depression [10]. Furthermore, anxiety, similar to depression, plays a key role in suicidality among PWE [11]. As a matter of fact, several studies have demonstrated the increased incidence of suicide in patients with psychiatric disorders and epilepsy. In a population-based case-control study in Denmark, having epilepsy and affective disorder increased the suicide risk by 32-fold, whereas having epilepsy and anxiety increased that risk by 11-fold [12]. Moreover, in a review of the literature, Jones et al. identified a life time average suicide rate of 12% in people with epilepsy compared to 1.1% to 1.2% in the general population [13].

The cause of the increased risk of both disorders in PWE is multifactorial. Genetic, neurochemical, anatomical, neurologic, and iatrogenic factors are among the most common studied etiologies [7]. However, for PWE, the perception that depression is a “normal” response to having a chronic condition has long been held, by both patients and physicians, but it is no longer acceptable or valid. Several studies have identified increasing risk of depression in PWE as compared to other chronic disease states [14, 15]. Other studies have also raised the possibilities of shared pathogenic mechanisms in both disorders that could account for that increased risk [16, 17].

In this study, and in order to test this “shared pathogenic mechanisms hypothesis,” we compared the rates of both depression and anxiety in PWE to another chronic brain disorder. Multiple sclerosis (MS) is a chronic debilitating neurological disorder that is known to be associated with increased incidence of both psychiatric diseases [18, 19]. We compared both rates to age and sex matched controls.

## 2. Materials and Methods

All patients seen in the epilepsy and MS clinics over the 6-month period, from September 2014 to April 2015, were asked to complete the Patient Health Questionnaire nine-item depression scale (PHQ-9) and Generalized Anxiety seven-item scale (GAD-7) questionnaires, and, if agreed to, they were asked to sit for a semistructured interview. In this study, we used the self-administered, English versions of PHQ-9 and GAD-7, and, for the few Arabic speaking patients attending our clinic, we used the Arabic validated versions of both tools [20–22]. We classified patients with PHQ-9 and GAD-7 scores ≥10 as having major depression and anxiety disorder, respectively. All patients completed the questionnaires before their assessment in the consultation room.

These tertiary referral clinics were established since 2006 to provide a comprehensive evaluations and treatments for all the residents in the Emirate of Abu Dhabi. We included patients of ages 18–65, with a confirmed diagnosis of epilepsy and MS. We included patients that were referred to the clinics for the first time, including patients with newly diagnosed epilepsy and MS, as well as patients who were seen for a follow-up visit. We excluded patients with progressive cognitive deficit that rendered patients incapable of signing the research consent form. The control group included a comparable number of healthy individuals, randomly selected from the general population and matched with the patients group for age and sex. We recruited controls from families of patients visiting the hospital clinics, college, and university students, employees of nonhealth related companies, and random people visiting the malls. All controls were enrolled if they did not have any chronic disease states. This study was approved by the local institutional and ethical review board.

## 3. Statistical Analysis

Gender and mean age were calculated for the three groups. For categorical variables, frequencies with percentages were calculated. In addition, odds ratios were calculated for the occurrence of depression and anxiety in PWE and those with MS. Statistical analysis was performed using chi-square test for categorical variables. The student *t*-test for continuous variables was used to compare the PHQ-9 and GAD-7 scores in PWE to those with MS. Significant *P* values were set at <0.05. Furthermore, since age and gender were not significantly different between the three groups, we did not include those two variables in the multivariate model.

## 4. Results

190 patients with a confirmed diagnosis of epilepsy were asked to enroll in the study, of which four patients have declined to participate. This leaves 186 patients who were recruited and agreed to participate, Table 1. Of these patients, 38 (20%) were seen in the clinic for the first time and 80% were seen for a follow-up visit. On the other hand, 163 patients with a confirmed diagnosis of MS, as per the modified McDonald criteria, were asked to enroll [23], of which 3 have declined to participate, leaving a total of 160 patients with MS who were enrolled. Of these, 16 (10%) were seen for the first time, and 90% were seen for a follow-up visit. The frequency of depression among the epilepsy group was 28.5%, significantly higher than the MS and matched controls (17.5, 16.1, resp.). On the other hand, the rates of anxiety among PWE were 26.2% which was not significantly higher than the MS and matched controls (20% and 15.6%, resp.), Table 2. When the rates of depression and anxiety among PWE were compared to the MS group, only rates of depression were statistically higher in PWE than in patients with MS (*P* = 0.016, OR = 1.9), whereas the rates of anxiety were not (*P* = 0.5), Table 3. When PHQ-9 and GAD-7 scores were used as continuous variables, PHQ-9 score was significantly higher in PWE compared to patients with MS (PWMS) (means: 6.9 versus 5.3, *P* = 0.005). On the other hand, GAD-7 score was not significantly different between the two groups (6.1 versus 5.2, *P* = 0.13).

## 5. Discussion

To the best of our knowledge, our study is the first study that has attempted to compare the frequencies of both depression and anxiety in patients with epilepsy to another chronic brain disorders, in this case, MS. Previous studies have compared the rates of depression to other chronic medical conditions. Ettinger et al. [14] have utilized the household panel maintained by the National Family Opinion to study depression and QOL in persons with epilepsy and asthma and healthy controls. 1532 patients responded to the survey. PWE were significantly more likely to score in the depressed range on the Center for Epidemiological Studies Depression Scale (CES-D) (37%) than were those with asthma (28%) or healthy subjects (12%). In another cross-sectional study, Beghi et al. [15] compared depression severity across disease states in a study of epilepsy, type I diabetes mellitus, and community controls. Fifty-five patients with idiopathic or cryptogenic epilepsy were compared with age and sex matched subjects with type I diabetes or persons donating blood in the local medical clinic. Epilepsy subjects with any structural brain abnormality were excluded from the study, and only 37% reported a seizure within the past year, reflecting less form of severe epilepsy, similar to the study of Ettinger et al. Thirty-four percent of epilepsy patients scored in the depressed range compared with 27% of type I diabetes patients and 7% of blood donors. Our findings are in agreement with the above studies, with the exception that the comparator groups were patients similar to PWE, those with a chronic brain disease state.

The perception that depression is a “normal” response to having a chronic condition such as epilepsy is no longer valid. Instead, several emerging data have explored other multifactorial etiologies of depression in epilepsy. It appears that epilepsy and depression may share common pathogenic mechanisms involving decreased serotonergic, noradrenergic, dopaminergic, and GABAergic activity, which has been shown to take part in the kindling process of seizure foci, exacerbating seizure frequency in some animal models [16]. Studies of neurotransmitter activity in both epilepsy and depression suggest that the occurrence of one disorder may facilitate the development of the other, and vice versa [17]. Likewise, three case-control population-based studies further support these shared pathogenic mechanisms. They have shown that a history of depression was associated with several fold increased risk for developing new onset epilepsy among cases than among controls [24–26]. These epidemiological studies further support the point towards the existence of common pathogenic mechanisms operant in both conditions which facilitate the development of one disorder in the presence of the other.

Interestingly, and despite the higher rates of both depression and anxiety in our PWE when compared to PWMS, only depression was significantly different in PWE, whereas anxiety was not. This finding further strengthens this strong relationship between both epilepsy and depression and probably further supports the shared pathogenic mechanisms of the two disorders. We chose MS as a comparator group, as MS is an example of a chronic debilitation brain disorder, and similar to patients with epilepsy it is highly associated with increased risk of depression and anxiety. On the other hand, we realize the difficulties of comparing point prevalence between both disorders, given the heterogeneity of the two populations and the multiple sociodemographic and disease related factors that were not accounted for in this study. We also acknowledge the limitations of our study, being a single center and relatively a small sample study. In addition, we have looked at the point prevalence rather than the life time prevalence rates of both psychiatric disorders, although it is unlikely that making the comparisons utilizing the life time prevalence would have yielded different results.

## 6. Conclusion

Our results show that depression and anxiety are probably more frequently encountered in patients with epilepsy when compared to other chronic neurological disease states. However, when comparing the rates of both psychiatric disorders in both chronic brain disorders, depression was significantly increased in patients with epilepsy compared to patients with MS. Larger, multicenter studies are needed to confirm our findings and to further support the shared pathogenic mechanisms between epilepsy and depression.

## Figures and Tables

**Table 1:** Patients with epilepsy and multiple sclerosis versus controls characteristics.

	MS patients (*n* = 160)	Epilepsy (*n* = 186)	Controls (*n* = 186)	*P*
Age (mean)	35	33.6	34.2	*P* > 0.1
Female	104 (65%)	105 (56.5%)	104 (55.9%)	*P* > 0.1
Male	48 (35%)	81 (43.5%)	82 (44.1%)	

**Table 2:** Rates of depression and anxiety among PWE, MS, and control groups.

Disorder	Epilepsy group *N* (%)	MS *N* (%)	Control *N* (%)	*P* value
Depression				
Present	64 (28.7%)	28 (17.5%)	26 (16.25%)	0.018
Absent	114 (71.3%)	132 (82.5%)	134 (83.75%)	
Anxiety				
Present	42 (26.2%)	32 (20%)	25 (15.625%)	0.015
Absent	118 (73.3%)	128 (80%)	135 (84.43%)	

**Table 3:** Comparative Rates of depression in PWE compared to PWMS.

Disorder	Epilepsy group *N* (%)	MS *N* (%)	OR (95% CI)	*P* value
Depression				
Present	64 (28.7%)	28 (17.5%)	1.9 (1.114–3.06)	0.05
Absent	114 (71.3%)	132 (82.5%)		
Anxiety				
Present	42 (26.2%)	32 (20%)	1.42 (0.62–2.98)	0.5
Absent	118 (73.3%)	128 (80%)		
